# Understanding Mental Health App Use Among Attendees of Primary Health Care in Taif, Saudi Arabia

**DOI:** 10.1192/bjo.2025.10465

**Published:** 2025-06-20

**Authors:** Sawsan Alkhammash, Mugtaba Osman

**Affiliations:** 1 Family Medicine Department, Prince Mansour Community Hospital, Taif, Saudi Arabia; 2 Mental Health Department, Prince Mansour Community Hospital, Taif, Saudi Arabia

## Abstract

**Aims:** Mental health apps are increasingly available and accessible to the public. Global research indicated variable rates of use among people with main barriers identified are cost, privacy concerns, and difficulty of use. Little is known about prevalence and barriers of use of mental health apps in Saudi Arabia.

**Methods:** Descriptive questionnaire-based cross-sectional survey of a sample of Saudi adult population. We adopted a multiple logistic regression modelling of data to evaluate the impact of potential barriers and facilitators on use of mental health apps.

**Results:** The survey included (n=636) participants. The prevalence of use of mental health apps was (n=80, 12.6%), with only (n=32, 40%) finding them useful. Younger age, females, separated marital status, students, history of mental illness, taking psychiatric medications, attending psychiatric services, seeing a psychologist (offline and online), and chatting to psychiatric patients online were all associated with unadjusted increase in use of mental health apps. However, the adjusted impact on use of mental health apps was significant only for those using psychiatric medications (odds ratio (OR)=0.1289, p=0.0243), individuals who requested online psychology intervention (OR=7.9866, p<0.00001), individuals who believed in costliness of mental health apps (OR=2.9358, p=0.00034) or difficulty using them (OR=4.1875, p=0.0002). Stigma and privacy concerns were not statistically impactful on use of mental health apps.

**Conclusion:** Use of mental health apps is very low among Saudi patients. Those who use mental health apps remain sceptical of their therapeutic values and report concerns in terms of difficulty to use them and their cost-effectiveness. Design of effective, readable, safe, and cheap mental health apps should be attempted by health educators and mental health professionals to engage Saudi patients in using mental health apps.

